# Pollakiuria: a rare and unique mode of revelation of silent dorsal syringomyelia in a young man—a case report

**DOI:** 10.1093/omcr/omaf149

**Published:** 2025-08-25

**Authors:** Richard Houeze, Mendinatou Agbetou Houessou, Alexandre D Faton, Salim Djaouga, Richmine Covi Zinsou, Eugénie Dansou, Martial Avoce, Constant K Adjien

**Affiliations:** University Neurology Clinic, National University Hospital Center Hubert, Koutoukou MAGA Littoral Department, Cotonou Cadjehoun District, Avenue Jean-Paul II, P.O. Box 01BP386, Benin; Department of Neurology, Faculty of Health Sciences, University of Parakou, Zongo II District, Parakou, Borgou Department, P.O. Box 123, Parakou, Republic of Benin; University Clinic for Physical Medicine and Rehabilitation, National University Hospital Center Hubert, Koutoukou MAGA, Littoral Department, Cotonou Cadjehoun District, Avenue Jean-Paul II, P.O. Box 01BP386, Benin; University Neurology Clinic, National University Hospital Center Hubert, Koutoukou MAGA Littoral Department, Cotonou Cadjehoun District, Avenue Jean-Paul II, P.O. Box 01BP386, Benin; University Neurology Clinic, National University Hospital Center Hubert, Koutoukou MAGA Littoral Department, Cotonou Cadjehoun District, Avenue Jean-Paul II, P.O. Box 01BP386, Benin; University Internal Medicine Clinic, National University Hospital Center Hubert, Koutoukou MAGA, Littoral Department, Cotonou Cadjehoun District, Avenue Jean-Paul II, P.O. Box 01BP386, Benin; University Neurology Clinic, National University Hospital Center Hubert, Koutoukou MAGA Littoral Department, Cotonou Cadjehoun District, Avenue Jean-Paul II, P.O. Box 01BP386, Benin; University Neurology Clinic, National University Hospital Center Hubert, Koutoukou MAGA Littoral Department, Cotonou Cadjehoun District, Avenue Jean-Paul II, P.O. Box 01BP386, Benin

**Keywords:** urinary urgency, Syringomyelia, Benin

## Abstract

Introduction: Syringomyelia is a rare pathology which is rarely revealed by urinary urgency. We report the case of a 20-year-old man with persistent urinary urgency and pollakiuria for two years, with no identifiable organic cause despite a thorough urological work-up. A transient episode of lower limb atony prompted spinal MRI, which revealed an isolated syrinx at the T6–T7 level, without associated Chiari malformation or conus medullaris involvement. The final diagnosis was vesico-sphincter dyssynergia with overactive bladder syndrome secondary to idiopathic thoracic syringomyelia. Conservative management, including anticholinergic therapy and psychological support, led to significant clinical improvement. Conclusion: As illustrated by our case, syringomyelia may present exclusively with urinary symptoms. However, such presentation is uncommon. This case highlights the importance of considering spinal cord pathology in the differential diagnosis of unexplained urinary symptoms, even in the absence of overt neurological deficits.

## Introduction

Pollakiuria refers to increased daytime urinary frequency without incontinence or dysuria. Syringomyelia most often presents with neurological symptoms. It is a rare disorder that infrequently presents as acute urinary retention. Urinary symptoms like detrusor overactivity and vesico-sphincter dyssynergia have been reported with syringomyelia [1].

Syringomyelia is traditionally considered a chronic and progressive condition with varied clinical manifestations [2]. Urinary symptoms, including pollakiuria can occur but are less common as initial manifestations.

Among urinary disturbances, pollakiuria is less common than difficulty voiding, retention, or incontinence. It is often associated to disruption of descending autonomic pathways controlling bladder function, leading to overactivity and increased urinary frequency. Micturition disorders occur in 9–25% of syringomyelia cases but remain uncommon. Some patients have refractory incontinence or voiding difficulties, with uncertain pathophysiology [3]. We report a case of syringomyelia revealed by urinary urgency.

## Case presentation

A 20-year-old man presented with a two-year history of persistent urinary urgency and pollakiuria, voiding 15 to 20 times per day. Clinical examination was unremarkable. Paraclinical investigations, including urine culture, pelvic ultrasound, and urinalysis, were all normal. Urodynamic testings were normal, leading to an initial diagnosis of a neurogenic bladder.

Due to persistent symptoms, he was referred to neurology. His medical history included daily moderate tension-like headaches in the cervico-occipital, bitemporal, and vertex areas, lasting about an hour, mainly in the evenings. No associated nausea, photophobia, or phonophobia were reported. Neurological exam revealed no motor or sensory deficits.

Hormonal workup (prolactin, FSH, LH, glucose) and brain MRI focusing on the thalamo-pituitary axis were normal. The preliminary diagnosis was tension-type headaches with a psychogenic overactive bladder.

**Figure 1:**
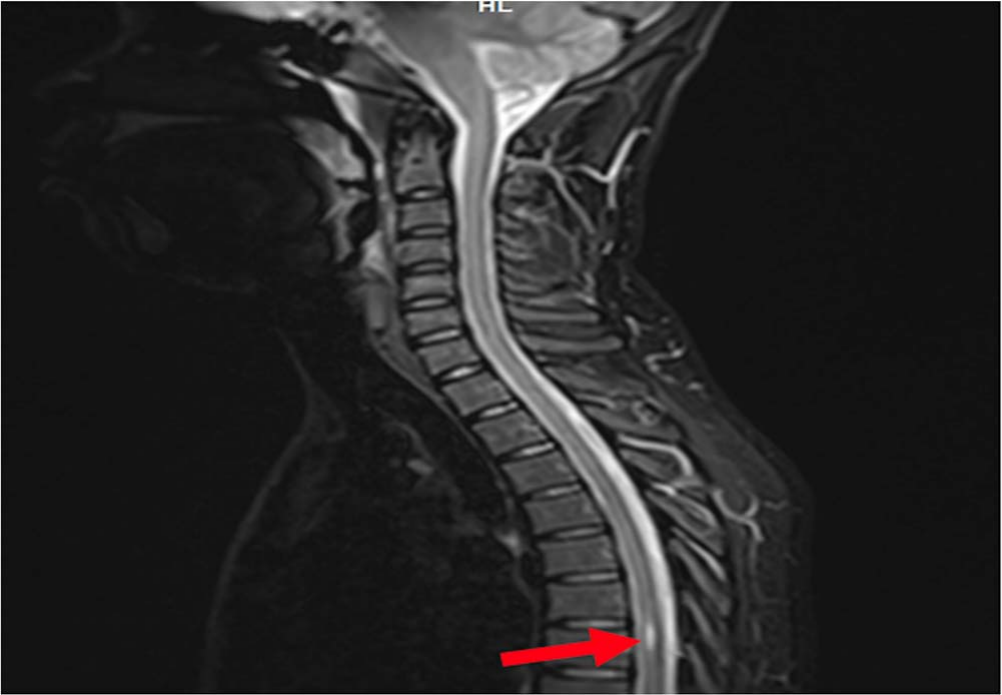
Sagittal T2-weighted MRI of the spinal cord performed for persistent pollakiuria in a 20-year-old man. A T2 hyperintense intramedullary lesion at the T6–T7 level is consistent with a syringomyelic cavity (red arrow) with no associated Chiari malformation.

**Figure 2:**
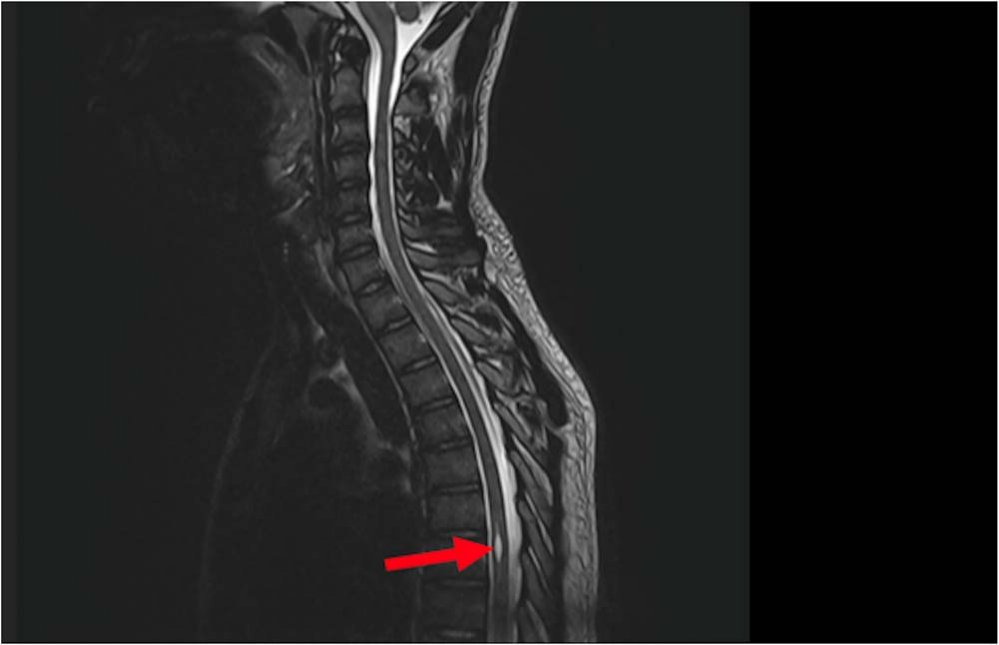
Sagittal T2-weighted MRI of the spinal cord performed for persistent pollakiuria in a 20-year-old man. A T2 hyperintense intramedullary lesion at the T6–T7 level is consistent with a syringomyelic cavity (red arrow) with no terminal conus abnormalities.

A transient episode of lower limb atony prompted further investigation. EEG was normal, but a full spinal MRI revealed a syrinx extending from T6 to T7, with no abnormalities in the conus medullaris or bulbomedullary junction (Figs 1 and 2).

No secondary cause was found. The final diagnosis was vesico-sphincter dyssynergia with overactive bladder due to idiopathic thoracic (T6–T7) syringomyelia, associated with tension-type headaches. Neurosurgical intervention was not indicated; clinical and radiological monitoring was recommended. The patient was started on solifenacin for overactive bladder, with favorable results. He also received psychological support.

## Discussion

Considering of syringomyelia in a patient with both pollakiuria and tention headache is difficult, especially with the absence of other neurological symptoms.

Urinary symptoms in syringomyelia may result from detrusor hypoactivity or detrusor hyperactivity with vesico-sphincter dyssynergia [1].

**Figure 3:**
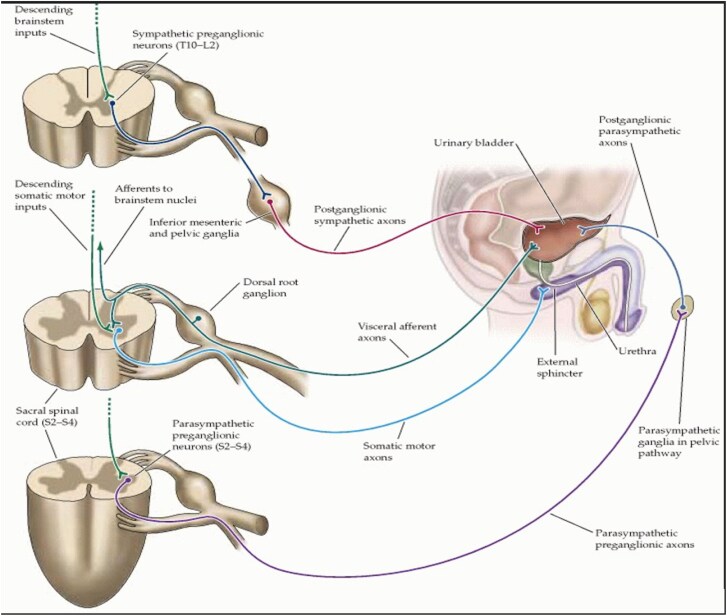
Neurophysiology of autonomic control of bladder function, Purves D (2001).

Bladder control involves sympathetic pathways (T10–L2) for detrusor relaxation and internal sphincter contraction, parasympathetic pathways (S2–S4) for detrusor contraction, and somatic pathways (pudendal nerve, S2–S4) for voluntary control of the external sphincter. A syrinx compressing these spinal tracts may interfere with inhibitory control or activate the micturition reflex, causing bladder overactivity and decreased capacity (Fig. 3) [4].

Previous case reports support early urinary symptoms as rare presentations of syringomyelia.

Pollakiuria is more often linked to urological or psychological causes. Its presentation as the sole symptom of a thoracic syrinx is extremely uncommon. Syringomyelia usually causes dissociated sensory loss, motor weakness, and pain, with urinary disturbances appearing later. Reports of isolated urinary symptoms as the first and only manifestation of syringomyelia are exceptionally rare.

Houang and al. described reversible urinary retention as the first symptom of syringomyelia from C5 to D11 with Chiari type I malformation in a 2-year-old child [5]. Amoiridis and al. reported urinary retention revealing extensive cervicothoracolumbar syringomyelia with Chiari I malformation [2]. Dysfunction of sacral parasympathetic neurons may underlie such cases [6, 7].

Lassalle reported a case of an 11-year-old with Charcot–Marie–Tooth disease type I and urinary symptoms; MRI revealed a syrinx from T11 to L1, and urodynamics showed detrusor overactivity [8].

Syringomyelia presenting solely with urinary symptoms is rare in both children and adults. Micturition symptoms are more common in advanced disease stages, and in most series, they appear years after initial neurological complaints. In a study of 14 patients with syringomyelia, urinary symptoms appeared an average of 5.3 years after neurological onset [3].

Lassalle described a [2-] year-old with urinary incontinence and a syrinx at T11–L2 [8]. Kothari and al. reported five pediatric patients with urinary disturbances as the primary complaint. Three had incontinence, and two had recurrent infections. All had thoracic syrinxes, with variable urodynamic results [9]. Iqbal reported a 1 [2-]year-old with enuresis and motor deficits due to a syrinx from C3 to T3 [10].

In most previously reported cases, urinary symptoms occurred alongside sensory or motor deficits. For example, one case described one patient with urinary retention and also paresthesias and mild motor signs, while another another case reported the case of one child with acute retention, cervicothoracic syrinx, and Chiari malformation along with subtle lower limb spasticity [5].

Our case is unique because pollakiuria was the only presenting symptom, without neurological signs.

Although our patient had clear urinary symptoms like urgency and pollakiuria, urodynamic studies (UDS) were normal. This may be due to intermittent or subclinical neurogenic bladder dysfunction, early selective disruption of autonomic sensory pathways by thoracic syringomyelia (T6–T7, in our case), compensation preserving detrusor function, or limited UDS sensitivity for subtle spinal involvement. These factors justified spinal imaging despite normal UDS.

This highlights the need to consider spinal cord pathology in patients with unexplained persistent urinary frequency, especially young individuals with normal urological evaluations. Early spinal MRI may enable prompt diagnosis and prevent neurological progression. The absence of sensorimotor symptoms could be due to the limited extent of the syrinx. In children and young adults, early attention to urinary complaints is essential. Misdiagnosis can lead to psychological distress and delayed intervention, leading to tension headaches requiring medical attention.

## Conclusion

As we have seen in our case, it's possible for syringomyelia to initially present with urinary symptoms alone. However, such presentation is uncommon. This unusual presentation underscores the need to consider spinal cord pathology in the differential diagnosis of persistent unexplained urinary frequency, especially in young patients with unremarkable urological evaluations. Early spinal MRI in such atypical scenarios could facilitate timely diagnosis and help prevent progressive neurological impairment.

## Data Availability

Not applicable.
